# Disease course after pregnancy in women with progressive multiple sclerosis symptoms

**DOI:** 10.1177/13524585251368248

**Published:** 2025-09-20

**Authors:** Jessica Shipley, Heidi N Beadnall, Paul G Sanfilippo, Dana Horakova, Cavit Boz, Alexandre Prat, Serkan Ozakbas, Tomas Kalincik, Izanne Roos, Ayse Altintas, Sara Eichau, Olga Skibina, Raed Alroughani, Francesco Patti, Masoud Etemadifar, Alessandra Lugaresi, Valentina Tomassini, Helmut Butzkueven, Anneke van der Walt, Vilija G Jokubaitis

**Affiliations:** Department of Neuroscience, School of Translational Medicine, Monash University, Melbourne, VIC, Australia; Department of Neurology, Alfred Health, Melbourne, VIC, Australia; Brain and Mind Centre, The University of Sydney, Sydney, NSW, Australia; Department of Neuroscience, School of Translational Medicine, Monash University, Melbourne, VIC, Australia; Department of Neurology and Center of Clinical Neuroscience, First Faculty of Medicine, Charles University and General University Hospital, Prague, Czech Republic; Department of Neurology, Medical Faculty, Karadeniz Technical University, Trabzon, Turkey; Centre hospitalier de l’Université de Montréal (CHUM), Université de Montréal, Montreal, QC, Canada; Izmir University of Economics, Medical Point Hospital, Izmir, Turkey; Multiple Sclerosis Research Association, Izmir, Turkey; Neuroimmunology Centre, Department of Neurology, Royal Melbourne Hospital, Melbourne, VIC, Australia; CORe, Department of Medicine, The University of Melbourne, Melbourne, VIC, Australia; Neuroimmunology Centre, Department of Neurology, Royal Melbourne Hospital, Melbourne, VIC, Australia; CORe, Department of Medicine, The University of Melbourne, Melbourne, VIC, Australia; Department of Neurology, School of Medicine and Koç University Research Center for Translational Medicine (KUTTAM), Koç University, School of Medicine, Istanbul, Turkey; Department of Neurology, Hospital Universitario Virgen Macarena, Seville, Spain; Department of Neurology, Alfred Health, Melbourne, VIC, Australia; Department of Neurology, Box Hill Hospital, Melbourne, VIC, Australia; Department of Neurosciences, Eastern Health Clinical School, Monash University, Melbourne, VIC, Australia; Division of Neurology, Department of Medicine, Amiri Hospital, Sharq, Kuwait; Department of Medical and Surgical Sciences and Advanced Technologies, G.F. Ingrassia, Catania, Italy; Multiple Sclerosis Unit, AOU Policlinico G Rodolico-San Marco, University of Catania, Catania, Italy; Department of Neurology, Dr. Etemadifar MS Institute, Isfahan University of Medical Sciences, Isfahan, Iran; Dipartimento di Scienze Biomedieche e Neuromotorie, Università di Bologna, Bologna, Italia; IRCCS Istituto delle Scienze Neurologiche di Bologna, Bologna, Italia; Institute for Advanced Biomedical Technologies (ITAB), Department of Neurosciences, Imaging and Clinical Sciences, University G. d’Annunzio of Chieti-Pescara, Chieti, Italy; Centre for Rehabilitation, Disability and Sport Medicine (CARES), Department of Neurosciences, Imaging and Clinical Sciences, Chieti, Italy; MS Centre, Clinical Neurology, SS Annunziata University Hospital, Chieti, Italy; Department of Neuroscience, School of Translational Medicine, Monash University, Melbourne, VIC, Australia; Department of Neurology, Alfred Health, Melbourne, VIC, Australia; Department of Neuroscience, School of Translational Medicine, Monash University, Melbourne, VIC, Australia; Department of Neurology, Alfred Health, Melbourne, VIC, Australia; Department of Neuroscience, School of Translational Medicine, Monash University, Melbourne, VIC, Australia; Department of Neurology, Alfred Health, Melbourne, VIC, Australia

**Keywords:** Multiple sclerosis, primary progressive multiple sclerosis, secondary progressive multiple sclerosis, pregnancy, disability, clinical outcomes

## Abstract

**Background:**

The impact of pregnancy on disease outcomes has not been characterised in women with progressive multiple sclerosis (MS) phenotypes. This study aimed to describe the clinical characteristics and disease course of women who experienced a pregnancy after a diagnosis of primary progressive MS (PPMS) or secondary progressive MS (SPMS).

**Methods:**

This multicentre observational cohort study utilised data from the international MSBase Registry extracted on 2 June 2024. Expanded Disability Status Scale (EDSS) scores of women with progressive MS were assessed up to 10 years postpartum and compared to those of propensity score–matched women with progressive MS without a pregnancy history.

**Results:**

In total, 138 women with 164 pregnancies were included in the study, comprising 75 women with PPMS and 63 with SPMS. Of these, 24 women with PPMS and 47 with SPMS had longitudinal peri-pregnancy EDSS assessments and were included in the analysis of disability scores. A history of pregnancy was not associated with a significant difference in long-term disability trajectories in women with either PPMS (estimate = −0.02; 95% confidence interval (CI) = −0.07 to 0.04) or SPMS (estimate = 0.00; 95% CI = −0.02 to 0.03).

**Conclusion:**

A history of pregnancy is not associated with a significant difference in long-term disability in women with progressive MS symptoms.

## Introduction

Multiple sclerosis (MS) is an inflammatory and degenerative disorder of the central nervous system that often affects women of reproductive age. Although a single disease entity, it is traditionally classified into distinct clinical phenotypes: relapsing-remitting MS (RRMS), secondary progressive MS (SPMS) and primary progressive MS (PPMS).^1^ RRMS is defined by episodic neurological relapses with relative stability between relapses. SPMS signifies the transition from RRMS to a phase of gradual disability progression independent of relapses, while PPMS presents with progressive disability accrual from symptom onset.^2^ Although the impact of pregnancy on disease outcomes has been extensively studied in RRMS, it has not been characterised in women with progressive symptoms.^3^

RRMS is most the common form of MS, affecting approximately 85% of individuals at diagnosis. It has a strong female predominance, with a 3:1 female-to-male ratio, and typically presents between the ages of 20 and 40 years.^4^ As a result, pregnancy is a frequent part of clinical care in the early years following an RRMS diagnosis. In contrast, SPMS typically emerges at least 10 to 15 years into the disease course, during later reproductive years or after the reproductive period.^5^ Similarly, PPMS has a mean age of onset closer to age 40 and affects men and women equally.^6,7^ Research on the impact of pregnancy has therefore primarily focused on women with RRMS, resulting in limited knowledge to guide family planning counselling for women with earlier-onset progressive disease.^8^

Pregnancy has well-established short-term effects on MS activity in women with RRMS, with relapse rates significantly decreasing during pregnancy, followed by disease reactivation in the first 3 months postpartum.^9,10^ However, the impact of pregnancy on disability progression is less certain. While most studies report no effect on disability progression,^11–17^ some suggest a protective^18–21^ or even negative impact.^22^ Although no studies have specifically examined the influence of pregnancy on women with progressive MS symptoms, a study of women with RRMS and more advanced disability found that higher preconception Expanded Disability Status Scale (EDSS) scores were associated with an increased odds of postpartum relapse and disability worsening in the early postpartum period.^23^ Limited research in women with PPMS has not found an association between general parity status and time to severe disability.^24,25^

This study aimed to describe the clinical characteristics and disease course of women who experienced a pregnancy after a diagnosis of progressive MS.

## Methods

### Study design and data collection

This was a multicentre retrospective observational cohort study using data from the MSBase Registry extracted on 2 June 2024. MSBase is an international registry that collects clinical data on patients with MS and other neuroimmunological conditions.^26^

### Study population

Patients were screened for study inclusion if they were female, aged 18–80 years, had clinically definite MS and had sufficient data recorded in the registry, including MS symptom onset date, MS phenotype and, if relevant, date of secondary progression. Women were included in the pregnant cohort if they had a documented pregnancy after the onset of either PPMS or SPMS, which was determined by the phenotype and dates recorded by MS specialist clinicians. Clinical characteristics and relapse activity were assessed in these women. For the analysis of disability trajectories, women were required to have at least one EDSS assessment both before and after pregnancy (‘baseline’). Only the first qualifying pregnancy was included in this analysis.

Women were selected for the non-pregnant cohort through a two-stage process. First, eligible women were identified if they had no documented pregnancies in the MSBase Registry, had PPMS or SPMS at baseline and had at least one EDSS score both before and after baseline. Baseline was assigned as the clinical visit date at which the individual’s age was closest to the mean age of the corresponding PPMS or SPMS cohort at pregnancy. Then, propensity score matching (PSM) was applied to identify women best matched to the pregnant cohort on key baseline covariates (see section ‘Statistical analysis’).

### Statistical analysis

Statistical analysis was performed using R (version 4.2.2; R Foundation for Statistical Computing, Vienna, Austria). Demographic and clinical characteristics of women with PPMS and SPMS were summarised using descriptive statistics. EDSS scores were plotted relative to pregnancy for each cohort, and median EDSS scores were modelled using smoothed curves fitted with locally weighted polynomial regression. A smoothing parameter of 0.5 was applied for the PPMS cohort and 0.3 for the SPMS cohort, representing the minimum parameters applicable based on data availability.

PSM was performed using the optimal matching method in the *MatchIt* package to select women in the non-pregnant cohort. This ensured balance with the pregnant cohort across key baseline covariates, including age, duration of progressive disease, EDSS score, disease-modifying therapy (DMT) use in the preceding year (binarised as ocrelizumab use for the PPMS cohort as the only DMT with evidence in this population^27^ and any DMT for SPMS), DMT epoch (before or after 1 January 2011) and relapse activity in the prior year. Several matching ratios were assessed, with a 2:1 ratio chosen to optimise both covariate balance and sample size. Balance was considered successful if the absolute standardised mean difference was < 0.1 for each covariate.

Differences in EDSS scores between pregnant and non-pregnant groups were evaluated using Mann–Whitney *U*-tests at yearly or grouped time intervals post-baseline, depending on sample size. Linear mixed-effects models were also used to assess the relationship between pregnancy history and EDSS scores over time, with time (years post-baseline) included as an interaction term to explore the effect of pregnancy history on EDSS scores over the observation period. In the PPMS cohort, the model was adjusted for post-baseline ocrelizumab exposure as a time-varying covariate (TVC), and in the SPMS cohort, high-efficacy DMT. High-efficacy DMT included alemtuzumab, anti-CD20 therapies, natalizumab, sphingosine-1-phosphate receptor modulators, cladribine, cyclophosphamide and autologous haematopoietic stem cell transplant. A random intercept was included for each patient to account for within-subject correlation across repeated measures. All analyses were two-tailed, with significance defined as *p* < 0.05.

Disability trajectories were also stratified by preconception DMT, defined as the DMT used in the year preceding pregnancy, or the DMT closest to pregnancy if more than one was used. In addition, the characteristics of women who experienced relapses during pregnancy or within the first 3 months postpartum were summarised.

### Standard protocol approvals, registrations and patient consents

The MSBase Registry has ethics approval or exemption from the local research ethics committee at each participating site, including the Alfred Health Human Research Ethics Committee, in line with local laws and regulations. All patients enrolled in the registry provided written informed consent in accordance with the Declaration of Helsinki.

### Data availability

MSBase is a data processor that stores data from individual Principal Investigators (PIs) who agree to share their datasets on a project-by-project basis. Qualified researchers may be granted data access at the discretion of each MSBase PI (contact the corresponding author for further information).

## Results

**Figure 1.**
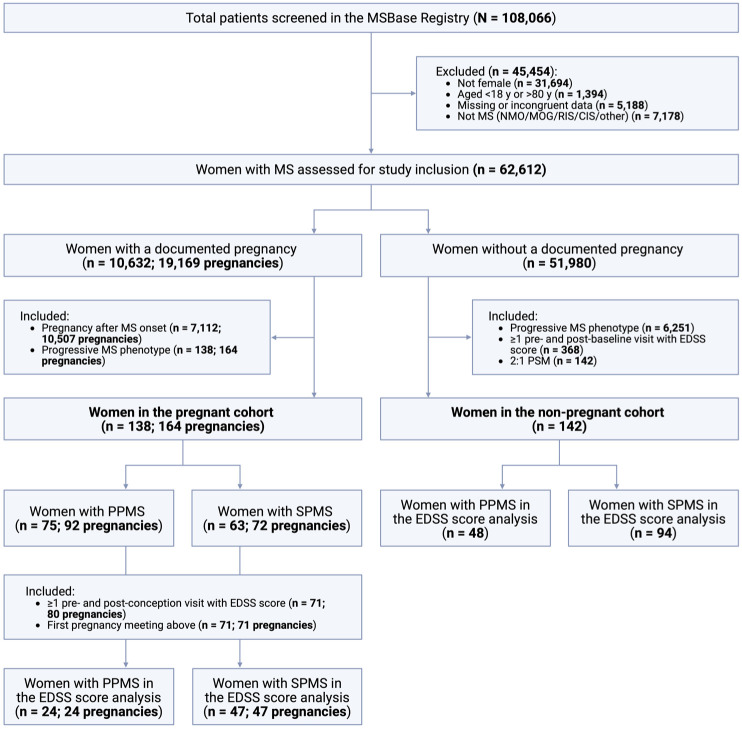
Flow diagram of participant inclusion and exclusion. CIS: clinically isolated syndrome; DMT: disease-modifying therapy; EDSS: Expanded Disability Status Scale; MOG: myelin-oligodendrocyte glycoprotein antibody-associated disease; NMO: neuromyelitis optica spectrum disorder; PPMS: primary progressive multiple sclerosis; PSM: propensity score matching; RIS: radiologically isolated syndrome; SPMS: secondary progressive multiple sclerosis.

We identified 7112 women with a documented pregnancy after MS symptom onset. Of these, 138 women had a progressive MS phenotype at the time of pregnancy, including 92 pregnancies in 75 women with PPMS and 72 pregnancies in 63 women with SPMS (Figure 1). Women in the pregnant cohort were from 47 centres across 17 countries (eFigure 1A–B in Supplement 1).

### Clinical characteristics

Table 1 summarises the clinical characteristics and treatment details associated with the included pregnancies. The median age at pregnancy was approximately 34 years for both women with PPMS (median 33.84 years, interquartile range (IQR) = 29.19–37.38) and SPMS (median = 34.54 years, IQR = 31.59–39.27). Women with PPMS were older at MS symptom onset (median = 28.56 years, IQR = 24.21–33.25) compared to those with SPMS (median = 23.66 years, IQR = 19.85–28.16), resulting in a shorter total disease duration at pregnancy for the PPMS cohort (median = 4.61 years, IQR = 2.34–7.27) compared to the SPMS cohort (median = 10.78 years, IQR = 8.13–13.48). However, the median duration of progressive disease was similar for women with SPMS (median = 3.24 years, IQR = 1.53–4.63). Pregnancy outcomes were comparable between the cohorts, with approximately 74% of pregnancies resulting in term births (⩾37 weeks’ gestation), 11% in preterm births (34–37 weeks’ gestation) and 15% in miscarriage or termination.

**Table 1.** Clinical characteristics and disease-modifying therapy use in women with progressive multiple sclerosis and pregnancy.

Characteristic	PPMS (*N* = 75 women with 92 pregnancies)	SPMS (*N* = 63 women with 72 pregnancies)
Age at MS symptom onset, median (IQR), y	28.56 (24.21–33.25)	23.66 (19.85–28.16)
Age at onset of secondary disease progression, median (IQR), y	–	31.73 (27.07–35.67)
Age at pregnancy, median (IQR), y	33.84 (29.19–37.38)	34.54 (31.59–39.27)
Disease duration at pregnancy, median (IQR), y^a^	4.61 (2.34–7.27)	10.78 (8.13–13.48)
Duration of progressive disease at pregnancy, median (IQR), y	4.61 (2.34–7.27)	3.24 (1.53–4.63)
Epoch of pregnancy, *n* (%)		
Modern (2011 or later)	41 (44.6)	28 (38.9)
Early (prior to 2011)	51 (55.4)	44 (61.1)
Pregnancy during peak COVID-19 years (2020–2021), *n* (%)	6 (6.5)	4 (5.6)
Pregnancy duration, median (IQR), w	39.00 (36.79–39.71)	39.00 (36.82–39.68)
Pregnancy outcome, *n* (%)		
Term birth	69 (75.0)	53 (73.6)
Preterm birth	10 (10.9)	8 (11.1)
Miscarriage or termination	13 (14.1)	11 (15.3)
Any preconception visit with EDSS score, *n* (%)	28 (30.4)	54 (75.0)
Months between preconception visit with EDSS score and conception, median (IQR)	3.12 (2.00–7.23)	5.32 (2.98–13.38)
Preconception EDSS score, median (IQR)^b^	3.5 (2.0–6.0)	5.0 (4.0–6.0)
Any postpartum visit with EDSS score, *n* (%)	88 (95.7)	69 (95.8)
Months between end of pregnancy and postpartum visit with EDSS score, median (IQR)	32.33 (8.85–106.77)	8.67 (2.89–27.27)
Postpartum EDSS, median (IQR)	4.5 (3.5–8.0)	6.0 (5.0–6.5)
Years of postpartum follow-up, median (IQR)	9.43 (4.01–18.81)	8.42 (4.17-13.08)
Documentation of breastfeeding, *n* (%)	25 (27.2)	17 (23.6)
DMT used in the preconception year, *n* (%)	17 (18.5)	46 (63.9)^c^
Anti-CD20 therapy	8 (8.7)^d^	4 (5.6)^e^
Natalizumab	4 (4.3)	7 (9.7)
Interferon	3 (3.3)	16 (22.2)
Glatiramer acetate	1 (1.1)	14 (19.4)
Cyclophosphamide	1 (1.1)	–
Fingolimod	–	5 (6.9)
High-efficacy DMT in the preconception year, *n* (%)	13 (14.1)	16 (22.2)
DMT used in the postpartum year, *n* (%)	12 (13.0)	31 (43.1)
Anti-CD20 therapy	6 (6.5)^f^	3 (4.2)^g^
Natalizumab	3 (3.3)	3 (4.2)
Glatiramer acetate	2 (2.2)	12 (16.7)
Interferon	1 (1.1)	6 (8.3)
Fingolimod	–	6 (8.3)
Dimethyl fumarate	–	1 (1.4)
High-efficacy DMT in the postpartum year, *n* (%)	9 (9.8)	12 (16.7)

DMT: disease-modifying therapy; EDSS: Expanded Disability Status Scale; IQR: interquartile range; PPMS: primary progressive multiple sclerosis; SPMS: secondary progressive multiple sclerosis; w: weeks; y: years.

a From the date of MS symptom onset.

b After the onset of progressive disease.

c One individual was not on ongoing DMT in the preconception year but had an autologous haemopoietic stem cell transplant 6.27 years prior to conception.

d Ocrelizumab (*n* = 8).

e Ocrelizumab (*n* = 3) and rituximab (*n* = 1).

f Ocrelizumab (*n* = 6).

g Ocrelizumab (*n* = 2) and rituximab (*n* = 1).

A significantly smaller proportion of women with PPMS (30.4%) had an EDSS score documented before pregnancy compared to those with SPMS (75.0%). The median preconception EDSS score was lower in the PPMS group (3.5, IQR = 2.0–6.0) than the SPMS group (5.0, IQR = 4.0–6.0). Nearly all women in both cohorts had a postpartum visit. However, the median time to the postpartum visit was substantially longer in women with PPMS (median = 32.33 months, IQR = 8.85–106.77) compared to SPMS (median = 8.67 months, IQR = 2.89–27.27). This time difference, and the discrepancy between preconception visit data, was not explained by reduced follow-up during the peak COVID-19 pandemic years, with a similar proportion of pregnancies between 2020 and 2021 in both groups.

Seventeen women with PPMS (18.5%) received DMT in the year prior to pregnancy, and 12 (13.0%) were treated with DMT in the postpartum year. Ocrelizumab was the most commonly used treatment in both periods. In the SPMS cohort, 46 women (63.9%) were prescribed DMT in the preconception year, most commonly interferon (22.2%). Thirty-one (43.1%) were prescribed DMT in the postpartum year, most often glatiramer acetate (16.7%).

### Disability scores relative to pregnancy

**Figure 2.**
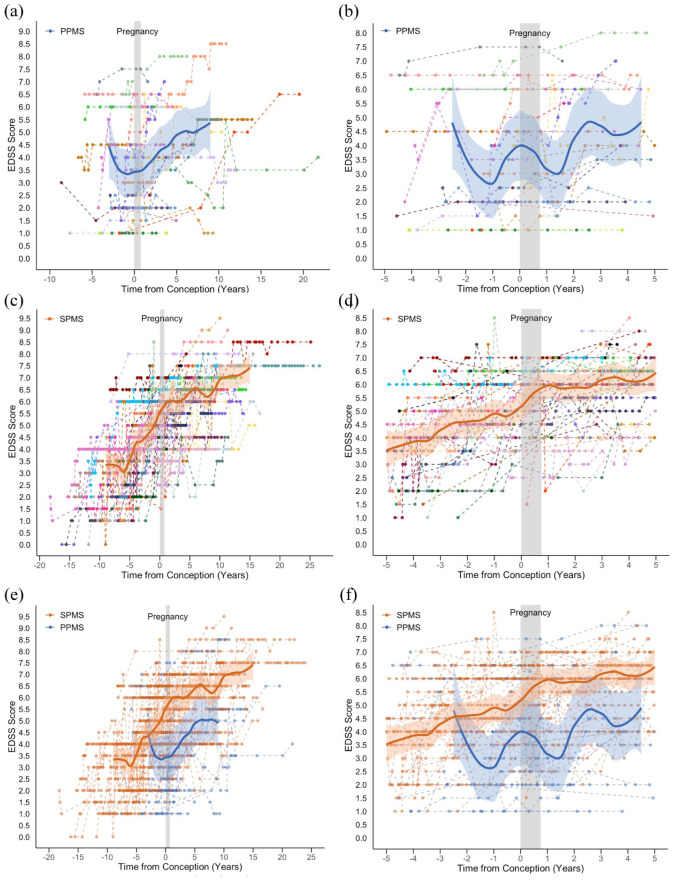
Disability scores relative to pregnancy in women with progressive multiple sclerosis. (a) Women with PPMS at all recorded time points; (b) women with PPMS at −5 to +5 years around pregnancy; (c) women with SPMS at all recorded time points; (d) women with SPMS at −5 to +5 years around pregnancy; (e) women with PPMS and SPMS at all recorded time points; (f) women with PPMS and SPMS at −5 to +5 years around pregnancy. The superimposed blue and orange lines show the Locally Estimated Scatterplot Smoothing (LOESS) of median EDSS scores after follow-up of a minimum of 10 individuals, with the shaded region representing the 95% confidence intervals for the predicted values of the LOESS smoothed curve. EDSS: Expanded Disability Status Scale; PPMS: primary progressive multiple sclerosis; SPMS: secondary progressive multiple sclerosis.

A total of 24 women with PPMS and 47 women with SPMS had EDSS scores recorded both before and after pregnancy. The total follow-up time for clinical visits with EDSS scores was 244.92 person-years for those with PPMS and 730.66 person-years for SPMS. The longitudinal EDSS scores for both cohorts are presented in Figure 2, including across all recorded time points and within 5 years before and after pregnancy. Raw median trajectories are shown in eFigure 2, and disability scores stratified by preconception DMT are included in eFigure 3.

In women with PPMS, there was significant variability in individual trajectories across the observed time points. In the plot of all recorded data, the median EDSS score model showed a brief negative gradient up to approximately 1 year before conception, followed by gradual disability progression over the subsequent 10 years (Figure 2(a)). In the −5 to +5-year time window, similar fluctuations in median EDSS scores were observed both before and after pregnancy (Figure 2(b)).

In the larger cohort of women with SPMS, disability progression was evident across the observation period and continued at a similar trajectory preconception and postpartum (Figure 2(c) and (d)). Comparing the PPMS and SPMS groups, median EDSS scores were higher in those with SPMS, likely reflecting the longer disease duration at pregnancy (Figure 2(e) and (f)).

### Disability scores in pregnant versus non-pregnant women

**Figure 3.**
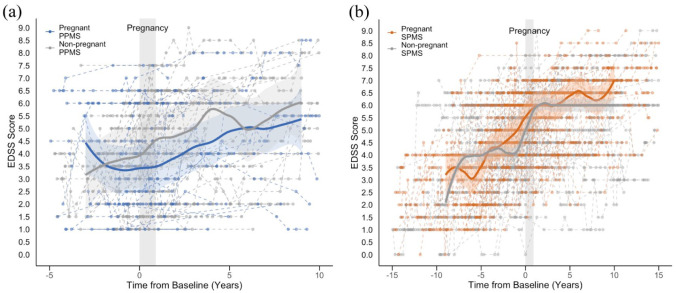
Disability scores in matched pregnant and non-pregnant women with progressive multiple sclerosis. (a) Disability scores of 24 pregnant women with PPMS relative to pregnancy and 48 women non-pregnant with PPMS relative to the matched baseline date and (b) disability scores of 47 pregnant women with SPMS relative to pregnancy and 94 non-pregnant women with SPMS relative to the matched baseline date. The superimposed blue, orange and grey lines show the Locally Estimated Scatterplot Smoothing (LOESS) of median EDSS scores after follow-up of a minimum of 10 individuals, with the shaded region representing the 95% confidence intervals for the predicted values of the LOESS smoothed curve. EDSS: Expanded Disability Status Scale; PPMS: primary progressive multiple sclerosis; SPMS: secondary progressive multiple sclerosis.

Using a 2:1 PSM ratio, 48 non-pregnant women with PPMS were matched to the 24 parous women with PPMS, and 94 non-pregnant women with SPMS were matched to the 47 pregnant women with SPMS. All baseline covariates were balanced between the groups (eTable 1A–B, eFigure 4A–B). Peri-pregnancy DMT use among the women included in the disability score analysis is detailed in eTable 2. Figure 3(a) and (b) illustrate the longitudinal EDSS scores of the pregnant and non-pregnant women with PPMS and SPMS, relative to pregnancy for the pregnant cohort and the matched baseline for the non-pregnant group. Median EDSS scores of the pregnant and non-pregnant cohorts significantly overlapped throughout the observation period for both phenotypes.

**Figure 4.**
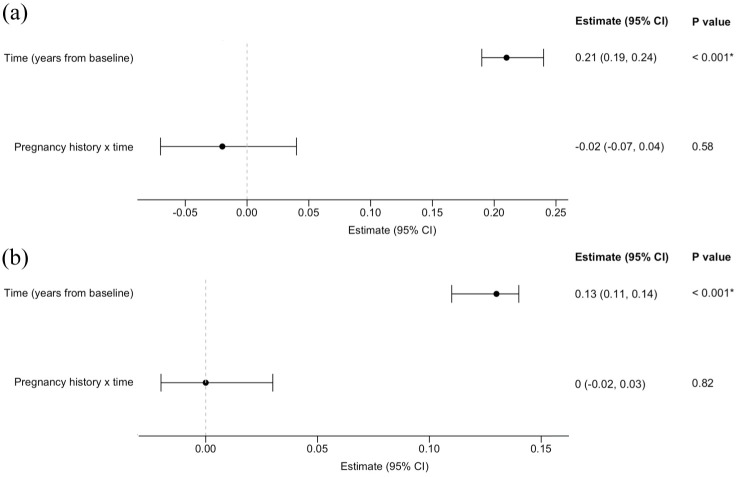
Association between pregnancy history and longitudinal disability scores in women with progressive multiple sclerosis. (a) Model for women with PPMS and (b) model for women with SPMS. Forest plots displaying results from linear mixed-effects models evaluating the interaction between pregnancy history and EDSS scores over a 10-year follow-up period post-baseline. The interaction term (pregnancy history × time from baseline) reflects differential EDSS trajectories by pregnancy status (see Table 2 and Table 3 for complete model results, including all covariates and coefficients). CI: confidence interval.

In the PPMS group, Mann–Whitney *U*-tests revealed no significant differences in EDSS scores between the pregnant and non-pregnant women across grouped time intervals (yearly intervals from baseline to 4 years and combined years 5 to 10) (eTable 3A). In addition, linear mixed-effects models, adjusted for ocrelizumab use as a TVC, found no statistically significant independent association between pregnancy history and EDSS scores over the 10-year follow-up period (estimate = −0.02; 95% confidence interval (CI) = −0.07 to 0.04; *p* = 0.58) (Figure 4(a); Table 2).

**Table 2.** Linear mixed-effects model of the interaction between pregnancy history and disability scores over time in women with primary progressive multiple sclerosis.

Variable	Estimate (95% CI)	df	*T*	*p*
Intercept	4.05 (3.48 to 4.61)	70.66	14.01	<0.001*
Pregnancy history	-0.57 (-1.55 to 0.41)	73.04	-1.13	0.26
Time (years from baseline)	0.21 (0.19 to 0.24)	606.56	14.87	<0.001*
Ocrelizumab exposure as a time-varying covariate	-0.18 (-0.41 to 0.06)	631.37	-1.45	0.15
Pregnancy history × time	-0.02 (-0.07 to 0.04)	605.92	-0.55	0.58

EDSS scores were modelled from 0 to 10 years post-baseline in women with PPMS with and without a history of pregnancy after MS onset. The intercept represents the estimated baseline EDSS score for the reference group – women without a history of pregnancy and not treated with ocrelizumab at baseline – with significance indicating the baseline EDSS score was significantly greater than 0. Pregnancy history was not independently associated with baseline disability. EDSS scores increased significantly over time, by an average of 0.21 points per year, consistent with gradual disability progression. Ocrelizumab exposure was associated with a non-significant trend towards lower EDSS scores. The interaction term (pregnancy history × time) was also non-significant, indicating no evidence that prior pregnancy altered the rate of disability progression over time. CI: confidence interval; df: degrees of freedom.

* *p* < 0.05.

**Table 3.** Linear mixed-effects model of the interaction between pregnancy history and disability scores over time in women with secondary progressive multiple sclerosis.

Variable	Estimate (95% CI)	df	*T*	*p*
Intercept	5.14 (4.84 to 5.41)	146.90	35.61	<0.001*
Pregnancy history	0.25 (-0.24 to 0.74)	148.80	1.01	0.31
Time (years from baseline)	0.13 (0.11 to 0.14)	1365.00	15.72	<0.001*
High-efficacy DMT exposure as a time-varying covariate	-0.20 (-0.32 to -0.09)	1417.00	-3.51	<0.001*
Pregnancy history × time	0.00 (-0.02 to 0.03)	1362.00	0.23	0.82

EDSS scores were modelled from 0 to 10 years post-baseline in women with SPMS with and without a history of pregnancy after MS onset. The intercept represents the estimated baseline EDSS score for the reference group – women without a history of pregnancy and not treated with high-efficacy DMT at baseline – with significance indicating the baseline EDSS score was significantly greater than 0. Pregnancy history was not independently associated with baseline disability. EDSS scores increased significantly over time, by an average of 0.13 points per year, consistent with gradual disability progression. High-efficacy DMT exposure was associated with significantly lower EDSS scores. The interaction term (pregnancy history × time) was non-significant, indicating no evidence that prior pregnancy altered the rate of disability progression over time. CI: confidence interval; df: degrees of freedom; DMT: disease-modifying therapy.

* *p* < 0.05.

For women with SPMS, there were no significant differences in EDSS scores between those with and without a history of pregnancy at yearly intervals from baseline to 10 years post-baseline (eTable 3B). Similarly, there was no significant association between pregnancy history and disability trajectories up to 10 years post-baseline (estimate = 0.00; 95% CI = −0.02 to 0.03; *p* = 0.82), with adjustment for high-efficacy DMT exposure (Figure 4(b) and Table 3).

### Relapse activity during and after pregnancy

Among the 92 pregnancies in women with PPMS, relapses were recorded during seven pregnancies (7.6%) (eTable 4A). Only one individual with a relapse during pregnancy received DMT in the preconception year. This patient was treated with natalizumab, which was discontinued 10 days before conception, and the relapse occurred at 10 weeks’ gestation. In the first 3 months postpartum, a relapse occurred in two women with PPMS (2.2%).

Of the 72 pregnancies in women with SPMS, relapses occurred during seven pregnancies (9.7%) (eTable 4B). Two of these women were treated with interferon, one with fingolimod and one with natalizumab. As stop dates for those on natalizumab and fingolimod were not recorded, it is uncertain whether these relapses occurred in the setting of therapy withdrawal. In the early postpartum period, relapses were recorded in seven women (9.7%).

## Discussion

A very small proportion of women in the large international MSBase Registry had a pregnancy after the onset of PPMS or SPMS. Among the 7112 women with a documented pregnancy following MS symptom onset, only 75 (1.1%) had PPMS and 63 (0.9%) had SPMS at conception. Fewer still had EDSS scores both before and after pregnancy. This highlights the rarity of pregnancy in women with progressive MS phenotypes and the challenges associated with studying the impact of pregnancy in these cohorts. In addition, the prevalence of progressive MS is likely underestimated in the registry due to DMT access and insurance implications, adding further challenges.^28^

Women with pregnancies after a PPMS diagnosis were significantly younger at symptom onset (median = 28.6 years) compared to general PPMS cohorts in the literature (mean = 41.2 years^6^). Similarly, women with SPMS had onset of initial relapsing-remitting symptoms approximately 10 years earlier than typically reported for RRMS (median = 23.7 years vs. 32 years).^29^ This explains the earlier onset of secondary progression at 31.7 years, although still significantly younger than the average age of SPMS conversion in MS registries.^28^ In addition, disability levels were high among women with SPMS, with a median preconception EDSS score of 5.0, indicating a cohort with severe MS with early progressive features. Most pregnancies in those with SPMS occurred in earlier treatment epochs (<2011), suggesting that early progression in this group may reflect delays to effective DMT.^30^ Furthermore, the documented progression may represent progression independent of relapse activity (PIRA) – which can occur during the relapsing-remitting phase – rather than typical secondary progression observed at older ages.^31^ While our cohorts are not representative of typical progressive populations – which is inherent to focusing on women of reproductive age – the study nonetheless offers insights into the impact of pregnancy on those with early-onset progressive symptoms.

Women in the PPMS cohort had notably less preconception visit data than those with SPMS. Only 30.4% of pregnancies in women with PPMS had a clinical visit with an EDSS score before pregnancy compared to 75% among those with SPMS. Shorter disease duration at conception could have contributed, but the median time to a postpartum visit with EDSS score was also significantly longer at 32.3 months in women with PPMS compared to 8.7 months in the SPMS group. As a result, the sample size of women with PPMS who had adequate data for analysis of disability trajectories relative to pregnancy was small, indicating the need for improved monitoring and/or documentation in this population. This might also reflect that patients with PPMS are less engaged with clinical services due to the limited therapeutic options available, especially prior to evidence for ocrelizumab.

Pregnancy did not influence the long-term disease course in women with either PPMS or SPMS. Although early postpartum disability scores could not be assessed due to limited timely follow-up in many women, gradual disability progression was observed across most of the follow-up period in both cohorts, with no apparent change in trajectory following pregnancy. While a transient improvement in median EDSS scores was noted prior to pregnancy in women with PPMS, the significance of this finding is uncertain, and this was followed by gradual disability progression consistent with the expected disease course. Moreover, when comparing matched pregnant and non-pregnant women with progressive MS, pregnancy history was not associated with a significant difference in EDSS scores or disability trajectories up to 10 years postpartum or post-baseline in either cohort. This is consistent with studies in RRMS that found no adverse effect of pregnancy on long-term disability outcomes^11–16^ and suggests that similar counselling is applicable for women with progressive symptoms.

Women with progressive MS were not immune to peri-pregnancy relapses. In the PPMS cohort, relapses occurred during 7.6% of pregnancies, and early postpartum relapses were documented after 2.6% of pregnancies. In SPMS, rates were 9.7% during pregnancy and 9.7% postpartum – slightly lower than in a contemporary RRMS cohort (11.8% and 13.6%, respectively).^32^ These findings support counselling women with progressive MS about the potential for peri-pregnancy relapses. While the relapses raise the possibility of phenotype misclassification, they also align with the evolving understanding of MS as a single disease continuum.^4,33,34^

## Limitations

This study had several limitations. Missing or incomplete registry data may have impacted the results. Specifically, some pregnancies may not have been recorded in the registry, potentially leading to misclassification of women in the non-pregnant cohort. In addition, the study relied on clinician-confirmed diagnoses of PPMS and SPMS. While there are no clinical criteria for determining the precise date of transition to SPMS, making this time point subject to individual clinician assessment,^5,35^ diagnoses were made by MS specialist neurologists. An additional important limitation of this study is the small sample size and low statistical power. Larger prospective studies are needed to further evaluate the effect of pregnancy on progressive MS, including the influence of peri-pregnancy relapses on disability progression. However, the rarity of pregnancies in this population presents considerable challenges.

## Conclusion

A history of pregnancy is not associated with a significant difference in long-term disability scores in women with progressive MS symptoms. However, in most patients with progressive MS, disability will continue to worsen after pregnancy, and patients should be informed about this anticipated course.

## Supplemental Material

sj-pdf-1-msj-10.1177_13524585251368248 – Supplemental material for Disease course after pregnancy in women with progressive multiple sclerosis symptoms Supplemental material, sj-pdf-1-msj-10.1177_13524585251368248 for Disease course after pregnancy in women with progressive multiple sclerosis symptoms by Jessica Shipley, Heidi N Beadnall, Paul G Sanfilippo, Dana Horakova, Cavit Boz, Alexandre Prat, Serkan Ozakbas, Tomas Kalincik, Izanne Roos, Ayse Altintas, Sara Eichau, Olga Skibina, Raed Alroughani, Francesco Patti, Masoud Etemadifar, Alessandra Lugaresi, Valentina Tomassini, Helmut Butzkueven, Anneke van der Walt and Vilija G Jokubaitis in Multiple Sclerosis Journal

sj-pdf-2-msj-10.1177_13524585251368248 – Supplemental material for Disease course after pregnancy in women with progressive multiple sclerosis symptoms Supplemental material, sj-pdf-2-msj-10.1177_13524585251368248 for Disease course after pregnancy in women with progressive multiple sclerosis symptoms by Jessica Shipley, Heidi N Beadnall, Paul G Sanfilippo, Dana Horakova, Cavit Boz, Alexandre Prat, Serkan Ozakbas, Tomas Kalincik, Izanne Roos, Ayse Altintas, Sara Eichau, Olga Skibina, Raed Alroughani, Francesco Patti, Masoud Etemadifar, Alessandra Lugaresi, Valentina Tomassini, Helmut Butzkueven, Anneke van der Walt and Vilija G Jokubaitis in Multiple Sclerosis Journal
