# Supplementary material for: Disease course after pregnancy in women with progressive multiple sclerosis symptoms
Source: Mult Scler. 2025 Sep 20;31(12):1439–51. doi: 10.1177/13524585251368248 (PMC12547043; doi:10.1177/13524585251368248)
Supplement: sj-pdf-1-msj-10.1177_13524585251368248 – Supplemental material for Disease course after pregnancy in women with progressive multiple sclerosis symptoms [file sj-pdf-1-msj-10.1177_13524585251368248.pdf]

Supplement 1

eFigure 1A. Countries of women with primary progressive multiple sclerosis

eFigure 1B. Countries of women with secondary progressive multiple sclerosis

eFigure 2. Disability scores around pregnancy in women with progressive multiple sclerosis with raw median lines

eFigure 3. Disability scores around pregnancy in women with progressive multiple sclerosis by preconception disease-modifying therapy

eTable 1A. Balance of covariates between pregnant and non-pregnant cohorts with primary progressive multiple sclerosis

eTable 1B. Balance of covariates between pregnant and non-pregnant cohorts with secondary progressive multiple sclerosis

eFigure 4A. Covariate balance between pregnant and non-pregnant cohorts with primary progressive multiple sclerosis before and after 2:1 propensity score matching

eFigure 4B. Covariate balance between pregnant and non-pregnant cohorts with secondary progressive multiple sclerosis before and after 2:1 propensity score matching

eTable 2. Peri-pregnancy disease-modifying therapy use in the subset of women in the EDSS score analysis

eTable 3A. Comparison of EDSS scores between pregnant and non-pregnant women with primary progressive multiple sclerosis

eTable 3B. Comparison of EDSS scores between pregnant and non-pregnant women with secondary progressive multiple sclerosis

eTable 4A. Characteristics of women with primary progressive multiple sclerosis and gestational or early postpartum relapse

eTable 4B. Characteristics of women with secondary progressive multiple sclerosis and gestational or early postpartum relapse

eFigure 1A. Countries of women with primary progressive multiple sclerosis

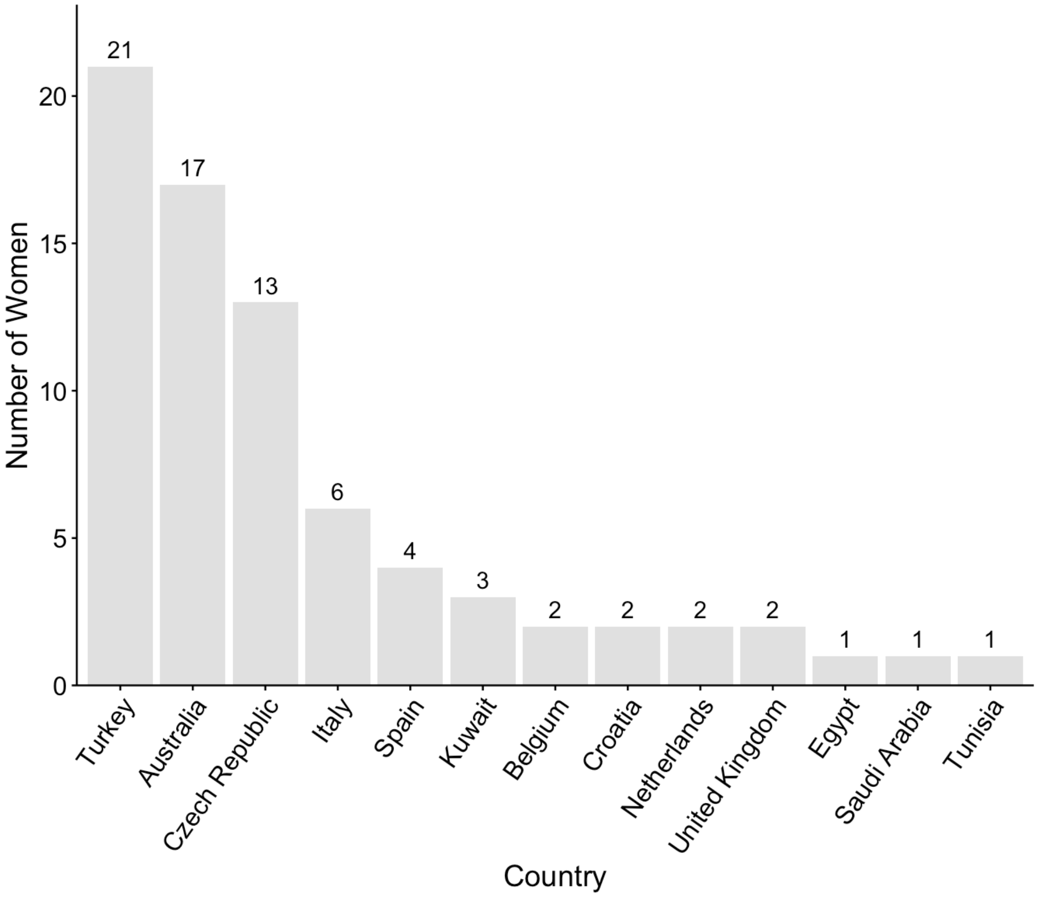

N = 75 women in the PPMS cohort

eFigure 1B. Countries of women with secondary progressive multiple sclerosis

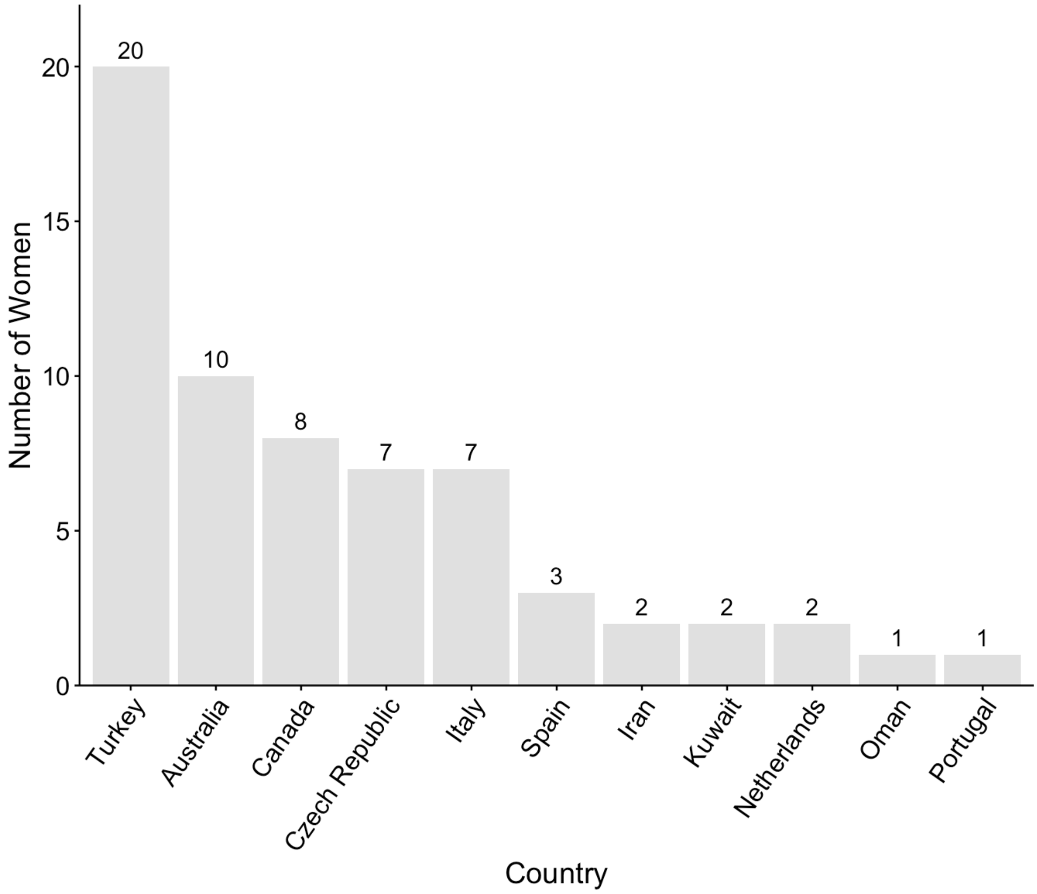

N = 63 women in the SPMS cohort

eFigure 2. Disability scores relative to pregnancy in women with progressive multiple sclerosis with raw median lines

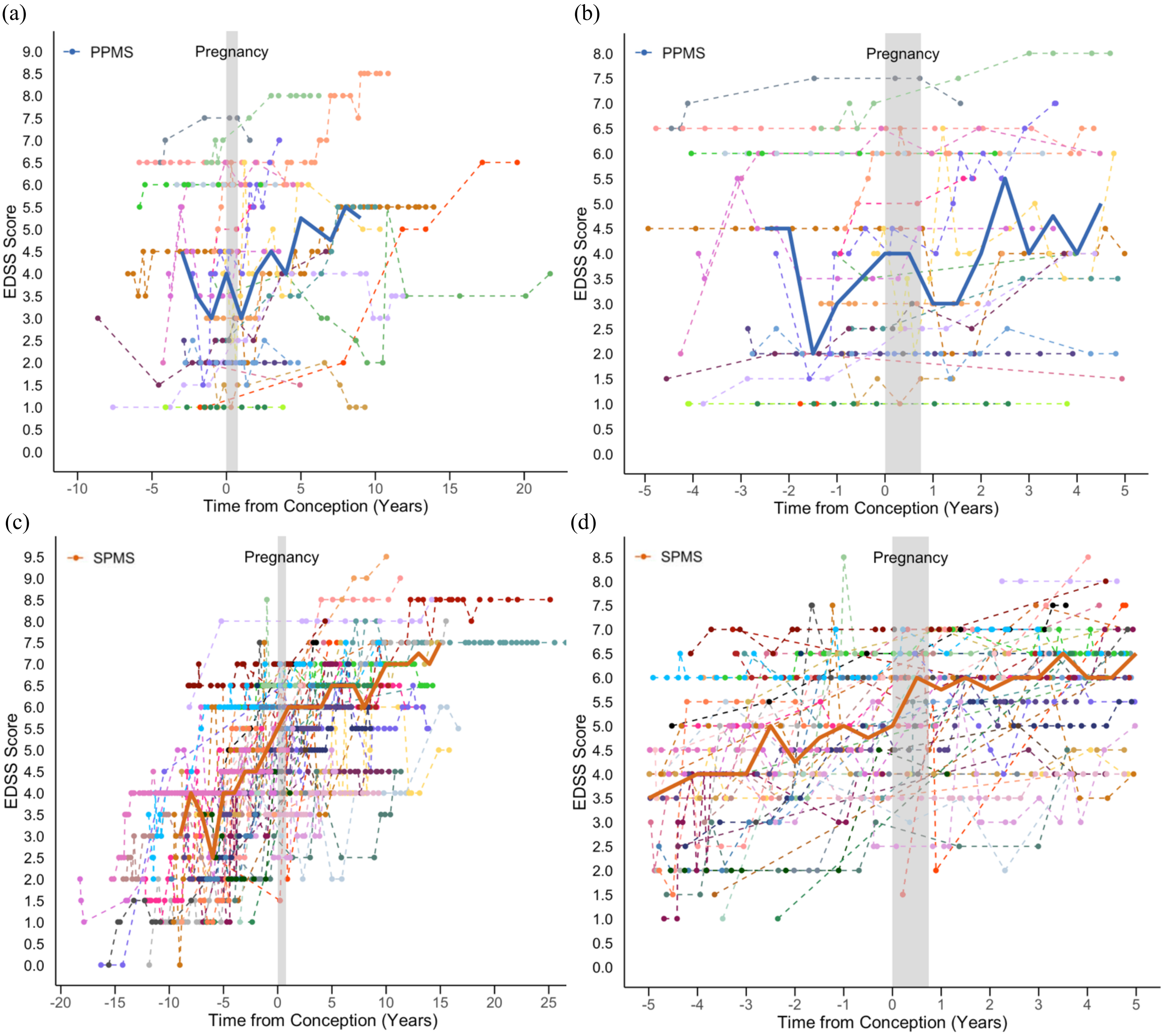

The superimposed blue and orange lines show the raw median EDSS scores after follow-up of a minimum of 10 individuals. (a) Women with PPMS at all recorded time points; (b) women with PPMS at -5 to +5 years around pregnancy; (c) women with SPMS at all recorded time points; (d) women with SPMS at -5 to +5 years around pregnancy. EDSS: Expanded Disability Status Scale; PPMS: primary progressive multiple sclerosis; SPMS: secondary progressive multiple sclerosis.

eFigure 3. Disability scores around pregnancy in women with progressive multiple sclerosis by preconception disease-modifying therapy

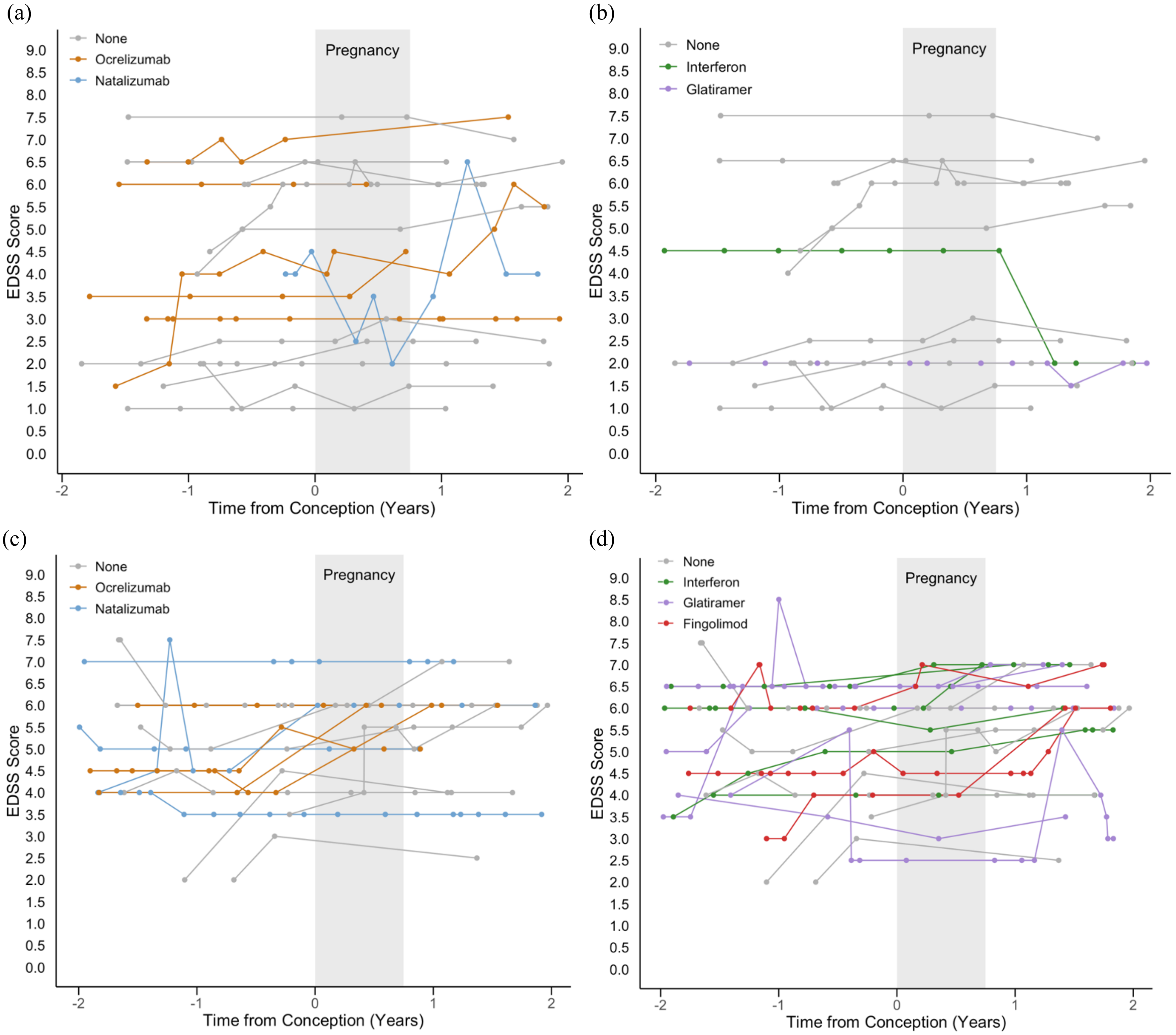

Plots of EDSS scores stratified by preconception DMT use in women with ≥2 EDSS scores in the window of -2 years to +2 years around pregnancy. (a), (b): Women with PPMS (n = 19); (c), (d): women with SPMS (n = 33). EDSS: Expanded Disability Status Scale; PPMS: primary progressive multiple sclerosis; SPMS: secondary progressive multiple sclerosis.

eTable 1A. Balance of covariates between pregnant and non-pregnant cohorts with primary progressive multiple sclerosis

**Covariate**	**Type**	**Unadj. mean (SD)**		**Unadj. SMD**	**Balance status**	**Adj. mean (SD)**		**Adj. SMD**	**Balance status**
		**Pregnant (N = 24)**	**Non-pregnant (N = 113)**			**Pregnant (N = 24)**	**Non-pregnant (N = 48)**		
Age at baseline	Cont	35.80 (6.13)	35.79 (1.04)	0.00	*Balanced*	35.80 (6.13)	35.72 (1.13)	0.03	*Balanced*
Disease duration at baseline	Cont	3.73 (2.22)	5.12 (2.00)	-0.66	Not balanced	3.73 (2.22)	3.84 (2.01)	0.10	*Balanced*
Baseline EDSS	Cont	6.40 (5.25)	8.64 (5.54)	-0.42	Not balanced	6.40 (5.25)	5.95 (3.86)	-0.02	*Balanced*
Ocrelizumab use in the pre-baseline year	Binary	-	-	0.31	Not balanced	-	-	0.04	*Balanced*
Epoch at baseline[a]	Binary	-	-	0.59	Not balanced	-	-	0.02	*Balanced*
Relapse in the pre-baseline year	Binary	-	-	-0.19	Not balanced	-	-	-0.08	*Balanced*

The first set of columns ("Unadjusted") compares baseline covariates between the pregnant PPMS cohort and the total pool of eligible non-pregnant women with PPMS prior to matching. The second set of columns ("Adjusted") shows the balance of covariates between pregnant and non-pregnant women after 2:1 propensity score matching. A variable was considered balanced if the absolute SMD for the covariate was less than 0.1. Adj.: adjusted; cont: continuous; EDSS: Expanded Disability Status Scale; PPMS: primary progressive multiple sclerosis; SD: standard deviation; SMD: standardised mean difference; SPMS: secondary progressive multiple sclerosis; unadj.: unadjusted; y: years.
[a]Binary variable for before or after 1 January 2011.

eTable 1B. Balance of covariates between pregnant and non-pregnant cohorts with secondary progressive multiple sclerosis

**Covariate**	**Type**	**Unadj. mean (SD)**		**Unadj. SMD**	**Balance Status**	**Adj. mean (SD)**		**Adj. SMD**	**Balance status**
		**Pregnant (N = 47)**	**Non-pregnant (N = 255)**			**Pregnant (N = 47)**	**Non-pregnant (N = 94)**		
Age at baseline	Cont	35.14 (5.41)	35.35 (1.51)	-0.05	*Balanced*	35.14 (5.41)	35.18 (1.62)	-0.01	*Balanced*
Disease duration at baseline[a]	Cont	4.79 (1.53)	5.95 (1.51)	-0.76	Not balanced	4.79 (1.53)	4.84 (1.52)	-0.04	*Balanced*
Baseline EDSS	Cont	3.84 (3.17)	4.29 (3.87)	-0.13	Not balanced	3.84 (3.17)	3.95 (3.70)	-0.03	*Balanced*
DMT use in the pre-baseline year	Binary	-	-	-0.05	*Balanced*	-	-	0.00	*Balanced*
Epoch at baseline[b]	Binary	-	-	-0.76	Not balanced	-	-	0.096	*Balanced*
Relapse in the pre-baseline year	Binary	-	-	-0.13	Not balanced	-	-	0.00	*Balanced*

The first set of columns ("Unadjusted") compares baseline covariates between the pregnant SPMS cohort and the total pool of eligible non-pregnant women with SPMS prior to matching. The second set of columns ("Adjusted") shows the balance of covariates between pregnant and non-pregnant women after 2:1 propensity score matching. A variable was considered balanced if the absolute SMD for the covariate was less than 0.1. Adj.: adjusted; cont: continuous; DMT: disease-modifying therapy; EDSS: Expanded Disability Status Scale; PPMS: primary progressive multiple sclerosis; SD: standard deviation; SMD: standardised mean difference; SPMS: secondary progressive multiple sclerosis; unadj.: unadjusted; y: years.
[a]Duration of secondary progressive disease at baseline.
[b]Binary variable for before or after 1 January 2011.

eFigure 4A. Covariate balance between pregnant and non-pregnant cohorts with primary progressive multiple sclerosis before and after 2:1 propensity score matching

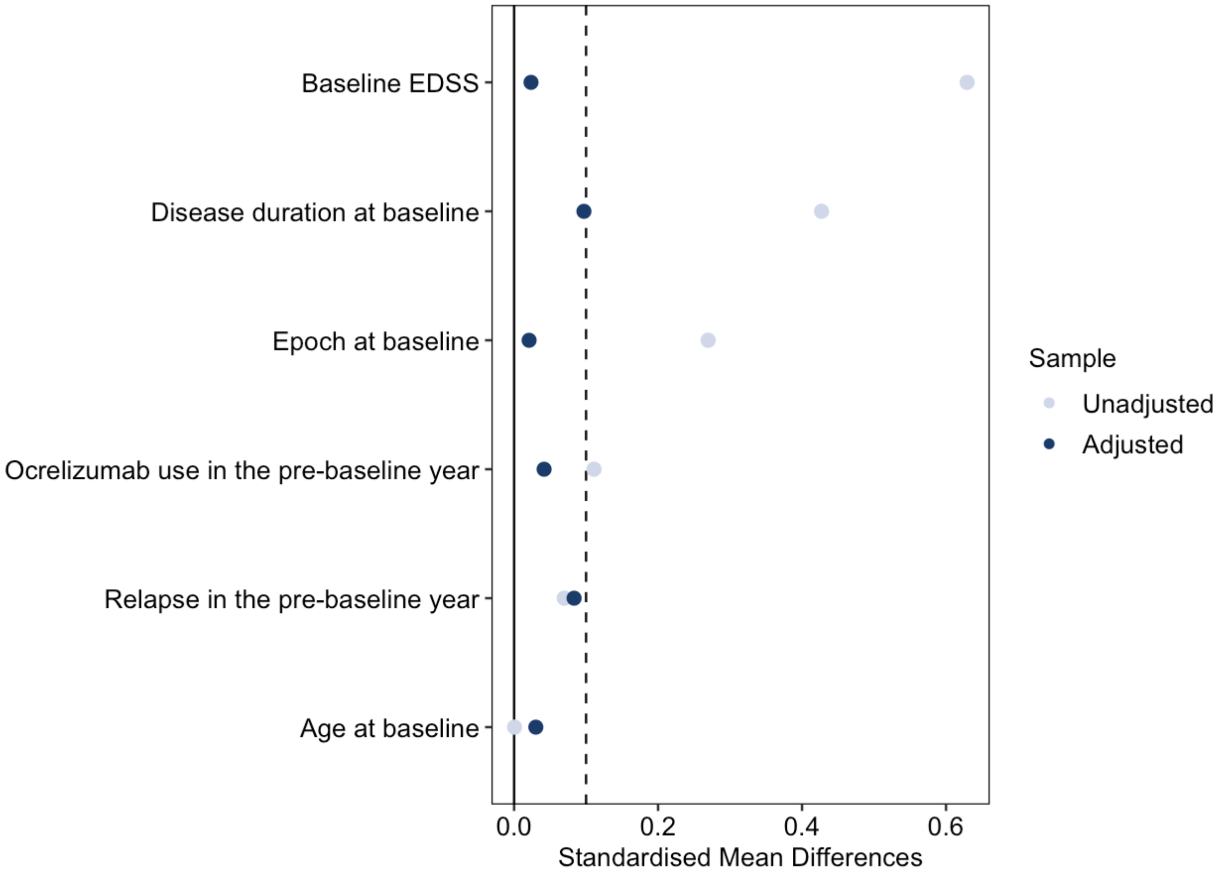

Love plot illustrating the absolute standardised mean differences for baseline covariates between the pregnant and non-pregnant groups before and after 2:1 propensity score matching. The dashed vertical line at 0.1 represents the commonly accepted threshold for acceptable covariate balance. EDSS: Expanded Disability Status Scale.

eFigure 4B. Covariate balance between pregnant and non-pregnant cohorts with secondary progressive multiple sclerosis before and after 2:1 propensity score matching

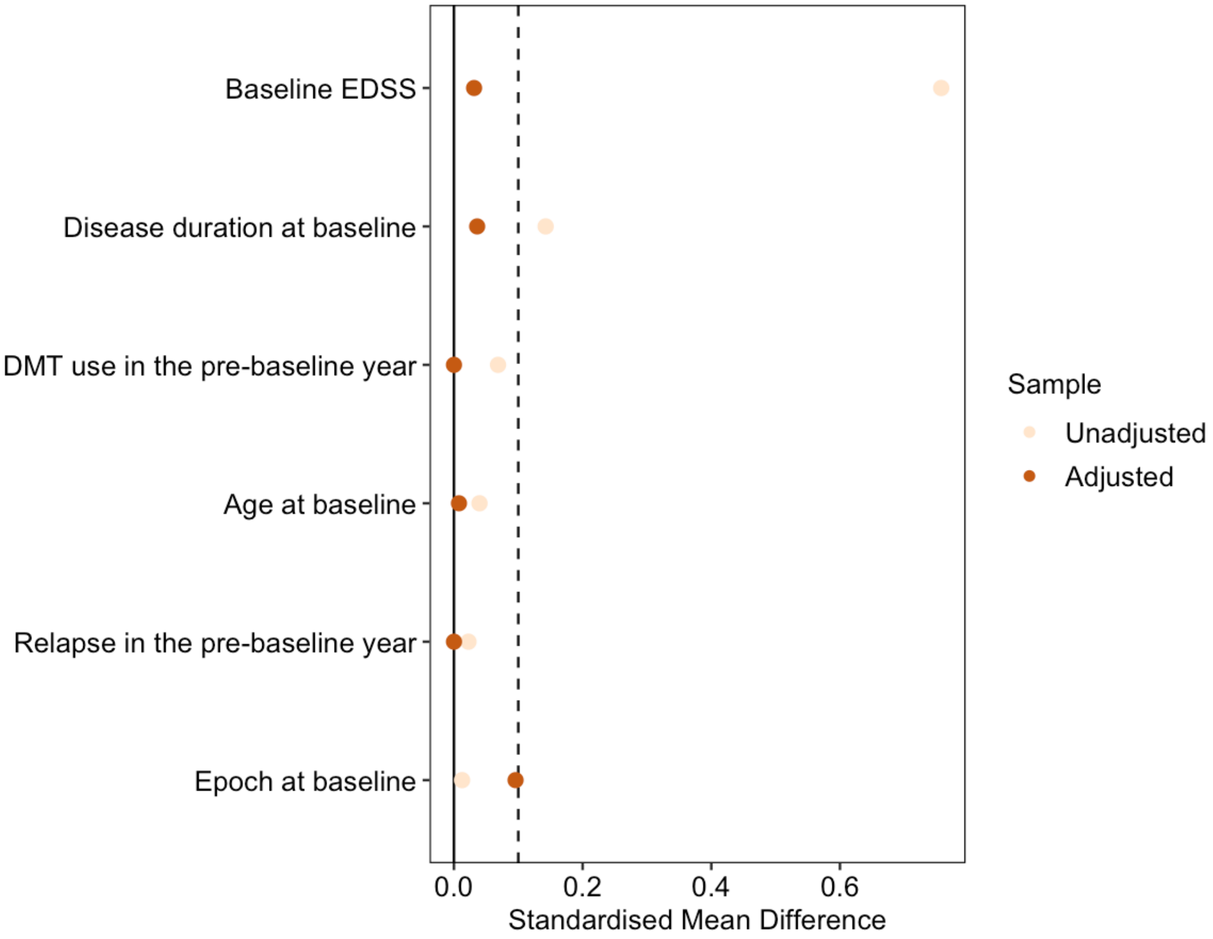

Love plot illustrating the absolute standardised mean differences for baseline covariates between the pregnant and non-pregnant groups before and after 2:1 propensity score matching. The dashed vertical line at 0.1 represents the commonly accepted threshold for acceptable covariate balance. DMT: disease-modifying therapy; EDSS: Expanded Disability Status Scale.

eTable 2. Peri-pregnancy disease-modifying therapy use in the subset of women with progressive multiple sclerosis in the EDSS score analysis

Characteristic	PPMS (N = 24)	SPMS (N = 47)
DMT used in the preconception year, n (%)	9 (37.5)	33 (70.2)[a]
Anti-CD20 therapy	5 (20.8)[b]	4 (8.5)[c]
Interferon	2 (8.3)	10 (21.3)
Glatiramer acetate	1 (4.2)	11 (23.4)
Natalizumab	1 (4.2)	5 (10.6)
Fingolimod	-	3 (6.4)
High-efficacy DMT in the preconception year, n (%)	6 (25.0)	12 (25.5)
DMT used in the postpartum year, n (%)	7 (29.2)	27 (57.4)
Anti-CD20 therapy	5 (20.8)[d]	3 (6.4)[e]
Glatiramer acetate	1 (4.2)	10 (21.3)
Natalizumab	1 (4.2)	3 (6.4)
Interferon	-	5 (10.6)
Fingolimod	-	5 (10.6)
Dimethyl fumarate	-	1 (2.1)
High-efficacy DMT in the postpartum year, n (%)	6 (25.0)	11 (23.4)

DMT: disease-modifying therapy; EDSS: Expanded Disability Status Scale; PPMS: primary progressive multiple sclerosis; SPMS: secondary progressive multiple sclerosis.

[a]One other individual was not on ongoing DMT in the preconception year but had an autologous haemopoietic stem cell transplant 6.27 years prior to conception.

[b]Ocrelizumab (n = 5).

[c]Ocrelizumab (n = 3), rituximab (n = 1).

[d]Ocrelizumab (n = 5).

[e]Ocrelizumab (n = 2), rituximab (n = 1).

eTable 3A. Comparison of disability scores between pregnant and non-pregnant women with primary progressive multiple sclerosis

**Years post baseline**	**Pregnant women, n**	**Non-pregnant women, n**	**Effect size *r***	***p***
0	19	46	-0.03	0.87
1	16	34	0.12	0.51
2	16	32	0.06	0.73
3	11	28	0.04	0.86
4	15	23	0.26	0.18
5 to 10	13	29	0.35	0.07

Calculated using the Mann–Whitney *U*-test. Interpretation of Cohen's *r* effect size: r = 0.10, 0.30, and 0.50 represent small, medium, and large effects, respectively.

eTable 3B. Comparison of disability scores between pregnant and non-pregnant women with secondary progressive multiple sclerosis

**Years post baseline**	**Pregnant women, n**	**Non-pregnant women, n**	**Effect size *r***	***p***
0	36	94	-0.09	0.43
1	31	70	-0.09	0.49
2	30	52	-0.21	0.11
3	31	47	-0.06	0.66
4	33	45	-0.01	0.96
5	26	38	-0.16	0.28
6	21	37	-0.16	0.32
7	20	38	-0.26	0.10
8	16	33	-0.23	0.19
9	21	27	-0.03	0.90
10	15	20	-0.17	0.39

Calculated using the Mann–Whitney *U*-test. Interpretation of Cohen's *r* effect size: r = 0.10, 0.30, and 0.50 represent small, medium, and large effects, respectively.

eTable 4A. Characteristics of women with primary progressive multiple sclerosis and gestational or early postpartum relapse

Patient	Timing of relapse, weeks[a]	Age at pregnancy, years	Pregnancy outcome	Relapse in the preconception year	Gestational relapse	DMT use in the preconception year	DMT washout period	DMT reinitiation postpartum	Time to DMT reinitiation, weeks	Steroid treatment
**Gestational relapse**										
Patient 1	9.57 (T1)	44.58	Preterm birth	N	-	None	-	-	-	Y
Patient 2	9.71 (T1)	37.37	Preterm birth	Y	-	Natalizumab	10 days	-	-	N
Patient 3	10.86 (T1)	21.54	Term birth	N	-	None	-	-	-	N
Patient 4	13.14 (T2)	31.08	Term birth	N	-	None	-	-	-	N
Patient 5	25.86 (T2)	35.78	Term birth	N	-	None	-	-	-	N
Patient 6	35.29 (T3)	32.33	Term birth	N	-	None	-	-	-	N
Patient 7	38.29 (T3)	27.04	Term birth	N	-	None	-	-	-	Y
**Relapse within the first 3 months postpartum**										
Patient 8	0.00	33.58	Term birth	N	N	None	-	None	-	N
Patient 9	6.43	36.33	Term birth	N	N	None	-	None	-	N

DMT: disease-modifying therapy; T1: first trimester of pregnancy: T2: second trimester of pregnancy; T3: third trimester of pregnancy; Y/N: yes, no.
[a]Weeks' gestation for relapse during pregnancy and weeks postpartum for relapse within the first 3 months postpartum.

eTable 4B. Characteristics of women with secondary progressive multiple sclerosis and gestational or early postpartum relapse

Patient	Timing of relapse, weeks[a]	Age at pregnancy, years	Pregnancy outcome	Relapse in the preconception year	Gestational relapse	DMT use in the preconception year	DMT washout period	DMT reinitiation postpartum	DMT reinitiation timing, weeks	Steroid treatment
**Gestational relapse**										
Patient 1	3.00 (T1)	26.61	Term birth	N	-	Interferon	7.26 months	-	-	N
Patient 2	7.29 (T1)	33.04	Termination at 10.14 weeks	N	-	Fingolimod	No stop date	-	-	Y
Patient 3	8.71 (T1)	35.83	Term birth	N	-	None	-	-	-	N
Patient 4	9.00 (T1), 21.71 (T2)	42.99	Term birth	N	-	None	-	-	-	N
Patient 5	11.00 (T1)	31.54	Term birth	N	-	Interferon	7.52 months	-	-	Y
Patient 6	23.43 (T2)	41.97	Term birth	N	-	None	-	-	-	N
Patient 7	25.86 (T2), 32.43 (T3)	30.21	Preterm birth	N	-	Natalizumab	No stop date	-	-	N
**Relapse within the first 3 months postpartum**										
Patient 8	1.57	40.37	Term birth	N	N	Interferon	0	Fingolimod	9.43	Y
Patient 9	2.86	32.92	Termination at 8.71 weeks	Y	N	None	-	None	-	N
Patient 5	2.86	31.54	Term birth	N	Y	Interferon	7.52 months	None	-	Y
Patient 10	3.43	27.41	Term birth	Y	N	None	-	Natalizumab	2.53	Y
Patient 4	4.43	42.99	Term birth	N	Y	None	-	Interferon	9.30	N
Patient 11	4.57	26.54	Term birth	N	N	None	-	None	-	N
Patient 12	11.14	32.79	Term birth	N	N	None	-	None	-	Y

DMT: disease-modifying therapy; T1: first trimester of pregnancy: T2: second trimester of pregnancy; T3: third trimester of pregnancy; Y/N: yes, no
[a]Weeks' gestation for relapse during pregnancy and weeks postpartum for relapse within the first 3 months postpartum.
