# Supplementary material for: Disease course after pregnancy in women with progressive multiple sclerosis symptoms
Source: Mult Scler. 2025 Sep 20;31(12):1439–51. doi: 10.1177/13524585251368248 (PMC12547043; doi:10.1177/13524585251368248)
Supplement: sj-pdf-2-msj-10.1177_13524585251368248 – Supplemental material for Disease course after pregnancy in women with progressive multiple sclerosis symptoms [file sj-pdf-2-msj-10.1177_13524585251368248.pdf]

Supplement 2

MSBase Study Group Coinvestigators

Name	Academic Degree	Affiliation 1	Contribution
Rana Karabudak	MD	Department of Neurological Sciences, Faculty of Medicine, Yeditepe University, Istanbul, Turkey	Data contributor
Marc Girard	MD	Centre hospitalier de l'Université de Montréal (CHUM) and Universite de Montreal, Montreal, Canada	Data contributor
Pierre Duquette	MD	Centre hospitalier de l'Université de Montréal (CHUM) and Universite de Montreal, Montreal, Canada	Data contributor
Guillermo Izquierdo	MD	Department of Neurology, Hospital Universitario Virgen Macarena, Seville, Spain	Data contributor
Katherine Buzzard	MBBS, PhD	Department of Neurology, Box Hill Hospital, Melbourne, Australia	Data contributor
Oliver Gerlach	MD	Academic MS Center Zuyd, Department of Neurology, Zuyderland Medical Center, Sittard-Geleen, Netherlands	Data contributor
Murat Terzi	MD	Medical Faculty, 19 Mayis University, Samsun, Turkey	Data contributor
Michael Barnett	MBBS, PhD	Brain and Mind Centre, Sydney, Australia	Data contributor
Marta Vachova	MD	Department of Neurology, KZ a.s., Hospital Teplice	Data contributor
Marek Peterka	MD	Department of Neurology, Faculty of Medicine and University Hospital in Pilsen, Charles University,	Data contributor
Yolanda Blanco	MD	Center of Neuroimmunology, Service of Neurology, Hospital Clinic de Barcelona, Barcelona, Spain	Data contributor
Abdorreza Naser Moghadasi		Multiple Sclerosis Research Center, Neuroscience Institute, Tehran University of Medical Sciences, Tehran, Iran	Data contributor
Emanuele D'Amico	MD	Medical and Surgical Sciences, Universita di Foggia, Foggia, Italy	Data contributor
Jeannette Lechner-Scott	MD, PhD	Hunter Medical Research Institute, University Newcastle, Newcastle, Australia	Data contributor
Bhim Singhal	MD	Bombay Hospital Institute of Medical Sciences, Mumbai, India	Data contributor
Vahid Shaygannejad	MD	Isfahan University of Medical Sciences, Isfahan, Iran	Data contributor
Maria Pia Amato	MD	Department NEUROFARBA, University of Florence, Italy	Data contributor
Maria Di Gregorio	MD	Neurology Unit, University Hospital San Giovanni di Dio e Ruggi d'Aragona, Salerno, Italy	Data contributor
Daniele Spitaleri	MD	Azienda Ospedaliera di Rilievo Nazionale San Giuseppe Moscati Avellino, Avellino, Italy	Data contributor

Davide Maimone	MD	Centro Sclerosi Multipla, UOC Neurologia, Azienda Opsedaliera per l'Emergenza Cannizzaro, Catania, Italy	Data contributor
Matteo Foschi	MD	Department of Neuroscience, MS Center, Neurology Unit, S. Maria delle Croci Hospital, AUSL Romagna, Ravenna, Italy	Data contributor
Andrea Surcinelli	MD	Department of Neuroscience, MS Center, Neurology Unit, S. Maria delle Croci Hospital, AUSL Romagna, Ravenna, Italy	Data contributor
Bassem Yamout	MD	Neurology Institute and MS Center, Harley Street Medical Centre, Abu Dhabi, United Arab Emirates	Data contributor
Samia J. Khoury	MD	Nehme and Therese Tohme Multiple Sclerosis Center, American University of Beirut Medical Center, Beirut, Lebanon	Data contributor
Talal Al-Harbi	MD	Neurology Department, King Fahad Specialist Hospital-Dammam, Saudi Arabia	Data contributor
Nevin John	MBBS, PhD	Department of Medicine, School of Clinical Sciences, Monash University, Clayton, Australia	Data contributor
Mark Slee	BMBS, PhD	College of Medicine and Public Health, Flinders University, Adelaide, Australia	Data contributor
Guy Laureys	MD	Department of Neurology, University Hospital Ghent, Ghent, Belgium	Data contributor
Pierre Grammond	MD	CISSS Chaudière-Appalache, Levis, Canada	Data contributor
Francois Grand'Maison	MD	Neuro Rive-Sud, Quebec, Canada	Data contributor
Jiwon Oh	MD	St. Michael's Hospital, Toronto, Canada	Data contributor
Jana Libertinova	MD, PhD	Department of Neurology, Second Faculty of Medicine, Charles University and Motol University Hospital, Prague	Data contributor
Nevin Shalaby	MD	Cairo University, Kasr-Al-Ainy MS Clinic, Cairo, Egypt	Data contributor
Celia Oreja-Guevara	MD	Department of Neurology, Hospital Clinico San Carlos, Madrid, Spain	Data contributor
Stella Hughes	MD	Royal Victoria Hospital, Belfast, United Kingdom	Data contributor
Mario Habek	MD, PhD	Department of Neurology, University Hospital Center Zagreb, Zagreb, Croatia	Data contributor
Elisabetta Cartechini	MD	Neurology Unit, AST Macerata, Macerata, Italy	Data contributor
Abdullah Al-Asmi	MD	College of Medicine & Health Sciences, Sultan Qaboos University, Al-Khodh, Oman	Data contributor
Joana Guimarães	MD, PhD	Department of Neurology, Unidade Local de Saúde de São João, Porto, Portugal	Data contributor
Riadh Gouider	MD	Department of Neurology, LR 18SP03, Clinical Investigation Centre Neurosciences and Mental Health, Razi University Hospital, Tunis, Tunisia	Data contributor
Saloua Mrabet	MD	Department of Neurology, LR 18SP03, Clinical Investigation Centre Neurosciences and Mental Health, Razi University Hospital, Tunis, Tunisia	Data contributor

Aysun Soysal	MD	Bakirkoy Education and Research Hospital for Psychiatric and Neurological Diseases, Istanbul, Turkey	Data contributor
Canun Yücesan		Neurology Department, Ankara University Ibni Sina Hospital	Data contributor
Edgardo Cristiano	MD	Centro de Esclerosis Múltiple de Buenos Aires (CEMBA), Buenos Aires, Argentina	Data contributor
Cameron Shaw	MBBS	Neuroscience Department, Barwon Health, University Hospital Geelong, Geelong, Australia	Data contributor
Suzanne Hodgkinson	MBBS, PhD	Immune tolerance laboratory Ingham Institute and Department of Medicine, UNSW, Sydney, Australia	Data contributor
Justin Garber	MBBS, PhD	Department of Neurology, Westmead Hospital, Sydney, Australia	Data contributor
Bruce Taylor	MD, PhD	Royal Hobart Hospital, Hobart, Australia	Data contributor
Richard Macdonell	MD	Austin Health, Melbourne, Australia	Data contributor
Pamela McCombe	MBBS	Department of Neurology, Royal Brisbane Hospital, Brisbane, Australia	Data contributor
Jennifer Massey	MD	St Vincent's Hospital, Sydney, Australia	Data contributor
Vincent van Pesch	MD, PhD	Department of Neurology, Cliniques Universitaires Saint-Luc, Brussels, Belgium	Data contributor
Melissa Cambron	MD, PhD	Neurology Department, Az Sint-Jan Brugge, Bruges, Belgium	Data contributor
Simón Cárdenas-Robledo		Centro de Esclerosis Múltiple (CEMHUN), Departamento de Neurología, Hospital Universitario Nacional de Colombia Bogota, Colombia	Data contributor
Jose Antonio Cabrera-Gomez	MD	Centro Internacional de Restauracion Neurologica, Havana, Cuba	Data contributor
Radek Ampapa	MD	Nemocnice Jihlava, Jihlava, Czech Republic	Data contributor
Pavel Hradilek	MD, PhD	Department of Neurology, Faculty of Medicine, Ostrava University,	Data contributor
Zbysek Pavelek	MD, PhD	Department of Neurology, Faculty of Medicine and University Hospital Hradec Kralove, Charles University in Prague,	Data contributor
Ivana Stetkarova	MD, PhD,	Department of Neurology, Third Faculty of Medicine, Charles University in Prague and Hospital Kralovske Vinohrady,	Data contributor
Pavel Stourac	MD, PhD,	Department of Neurology, Masaryk University Brno and University Hospital, Brno	Data contributor
Nevin Shalaby	MD	Cairo University, Kasr-Al-Ainy MS Clinic, Cairo, Egypt	Data contributor
Ricardo Fernandez Bolaños	MD	Hospital Universitario Virgen de Valme, Seville, Spain	Data contributor
Jose Luis Sanchez-Menoyo	MD	Department of Neurology, Galdakao-Usansolo University Hospital, Osakidetza-Basque Health Service, Galdakao, Spain	Data contributor
Cristina Ramo-Tello	MD, PhD	Department of Neuroscience, Hospital Germans Trias i Pujol, Badalona, Spain	Data contributor

MSBase Foundation Centre Custodian	MD	Institute of Neuroscience Buenos Aires, Buenos Aires, Argentina	Data contributor
Claudio Solaro	MD	Neurology Unit, Galliera Hospital, Genova, Italy	Data contributor
MSBase Foundation Centre Custodian	MD	Institute of Neuroscience Buenos Aires, Buenos Aires, Argentina	Data contributor
Koen de Gans	MD	Groene Hart Ziekenhuis, Gouda, Netherlands	Data contributor
Maria Edite Rio	MD	Centro Hospitalar Universitario de Sao Joao, Porto, Portugal	Data contributor
Gregor Brecl Jakob		Department for Neurology, Department of Neurology, University Medical Centre Ljubljana, Slovenia	Data contributor
Bianca Weinstock-Guttman	MD	Department for Neurology, Jacobs MS center for treatment and research, United States	Data contributor
